# Constricted pathways: esophageal and laryngeal stenosis

**DOI:** 10.11604/pamj.2024.48.58.43863

**Published:** 2024-06-13

**Authors:** Jay Dinesh Bhanushali

**Affiliations:** 1 Department of Respiratory Medicine, Jawaharlal Nehru Medical College, Datta Meghe Institute of Higher Education and Research, Sawangi (Meghe), Wardha, Maharashtra, India

**Keywords:** Dysphagia, carcinoma, metastasis, tracheal stenosis, laryngeal oedema

## Image in medicine

A 39-year-old female presented to the emergency department with complaints of difficulty swallowing solids and liquids, along with cough and breathlessness on exertion for the past 30 days. The patient gave a history of carcinoma of the tongue for which she was operated and had received radiotherapy 2 years back. The patient was admitted for further evaluation and fibre optic flexible bronchoscopy showed significant laryngeal stenosis and the bronchoscope could not be negotiated further through the vocal cords. Esophageal stenosis was also noted, along with multiple rounded 0.5x0.5 cm mass lesions at the proximal esophageal opening. Biopsy was taken, which was suggestive of well-differentiated squamous cell carcinoma. Post-radiotherapy laryngeal and esophageal stenosis can occur as a complication of radiation therapy aimed at treating cancers in the head and neck region. Radiation therapy, while effective in targeting cancer cells, can also affect nearby healthy tissues, leading to inflammation, fibrosis, and reduced elasticity of the tissues in the throat and esophagus. Symptoms may include hoarseness, coughing, choking, difficulty swallowing, pain during swallowing, shortness of breath, and a sensation of something stuck in the throat. Treatment options may include speech therapy, dilation procedures, surgical intervention, or a combination of these approaches, depending on the severity and specific manifestations of stenosis.

**Figure 1:**
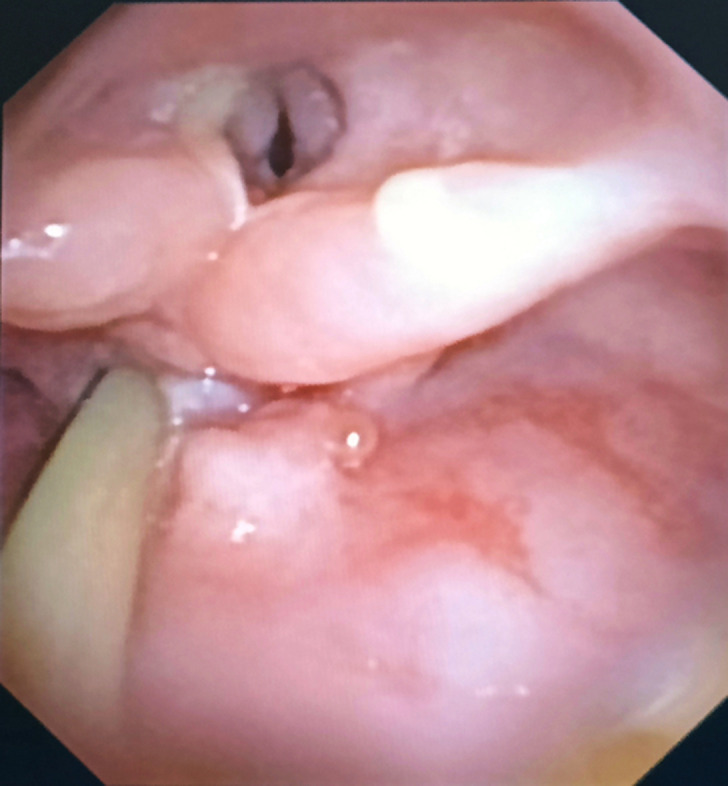
bronchoscopic view of oropharynx showing laryngeal stenosis and esophageal stenosis with a mass lesion with ryles tube in situ

